# Modeling foot-and-mouth disease dissemination in Rio Grande do Sul, Brazil and evaluating the effectiveness of control measures

**DOI:** 10.3389/fvets.2024.1468864

**Published:** 2024-10-25

**Authors:** Nicolas C. Cardenas, Francisco P. N. Lopes, Alencar Machado, Vinicius Maran, Celio Trois, Felipe Amadori Machado, Gustavo Machado

**Affiliations:** ^1^Department of Population Health and Pathobiology, College of Veterinary Medicine, North Carolina State University, Raleigh, NC, United States; ^2^Departamento de Defesa Agropecuária, Secretaria da Agricultura, Pecuária e Desenvolvimento Rural, Porto Alegre, Brazil; ^3^Laboratory of Ubiquitous, Mobile and Applied Computing (LUMAC), Polytechnic College of Federal University of Santa Maria, Santa Maria, Brazil; ^4^Department of Chemical Engineering, Rovira i Virgili University, Tarragona, Spain

**Keywords:** dynamical models, infectious disease control, epidemiology, transmission, targeted control, FMD (foot-and-mouth disease), simulation

## Abstract

**Introduction:**

Foot-and-mouth disease (FMD) affects multiple food-animal species and spreads rapidly among ungulate populations, posing significant challenges for disease control. Understanding the dynamics of FMD transmission and evaluating the effectiveness of control measures are critical for mitigating its impact. This study introduces a multiscale compartmental stochastic model to simulate FMD spread and assess countermeasures.

**Methods:**

We developed a model that integrates population dynamics, including births, deaths, and species-specific transmission dynamics, at both the between-farm and within-farm levels. Four scenarios were created to evaluate different control strategies: the base scenario included vaccinating 20 farms and depopulating four infected farms, while alternative scenarios increased vaccination and depopulation capacities or omitted vaccination altogether.

**Results:**

Our simulations showed that bovines were the most frequently infected species, followed by swine and small ruminants. After 10 days of initial spread, the number of infected farms ranged from 1 to 123, with 90.12% of simulations resulting in fewer than 50 infected farms. Most secondary spread occurred within a 25 km radius. An early response to control actions significantly reduced the time spent managing outbreaks, and increasing daily depopulation and vaccination capacities further enhanced control efforts.

**Discussion:**

Emergency vaccination effectively reduced the magnitude and duration of outbreaks, while increasing depopulation without vaccination also eliminated outbreaks. These findings highlight the importance of rapid response and capacity scaling in controlling FMD outbreaks, providing valuable insights for future decision-making processes in disease management.

## 1 Introduction

Foot-and-mouth disease (FMD) is an infectious disease in cloven-hoofed animals that affects multiple species, including bovine, swine, small ruminants, and wildlife (1). This disease can also impact the economies of affected countries. During the 2001 FMD epidemic in the U.K. and the Netherlands, more than 6.7 million animals were slaughtered, including healthy ones (preemptive culling) (2). In both outbreaks, multiple species were infected, including goats on mixed dairy-goat/veal-calf farms (2, 3), and there were additional costs to other sectors, such as tourism, with a total expenditure of approximately 2.7 to 3.2 billion euros (4). The official World Organisation for Animal Health (WOAH, 2022) database recorded more than 2,484,001 outbreaks in 80 countries from 2015 to 2023, showing that 73.43% of FMD cases were associated with cattle, 3.02% with swine, 14.38% with small ruminants, and 4.8% with buffaloes (5). In South America, no large outbreaks have been reported since 2001, when 2,027 farms in Uruguay were affected, with cattle and small ruminants being the predominant infected species (6), up to date, the most recent outbreaks reported happened in Colombia between 2017 and 2018 (7); since then no epidemics have been officially reported in America, despite Venezuela’s absence of official international status for FMD (8).

Despite substantial evidence that all susceptible species can contribute to significant FMD epidemics, response plans frequently focus on controlling the spread among cattle populations. This approach often overlooks the role of other domestic species (9). This makes it important to consider that the pathogenesis and transmission dynamics vary among species, given differences in viral loads needed to cause infection, variability in latency, and infection duration (10–13). For instance, infected swine shed more viral particles than cattle and sheep, historically resulting in widespread epidemics expected when infected (12). Thus, it is pivotal to consider such heterogeneity in transmission dynamics when modeling within and/or between-farm FMD dissemination (9). In the same vein, field observations and experimental trials have demonstrated the spread of FMD occurs between-farm transmission primarily occurs through direct contact among susceptible and infected animals (10, 14), and via indirect contact with fomites and long-distance transport of aerosols, a process known as spatial transmission (14).

Mathematical models have been widely used to investigate FMD epidemic propagation (15, 16). Although significant technical and computational advancements have been achieved, simplifications of complex dynamics are required because of computational costs or the lack of population data, such as details about herd structure (e.g., number of individuals, number born alive) or animal movements, for example (15). The most common model simplification involves limiting the dynamics to a single species (4, 17, 18). Despite the different applications and efforts in modeling FMD, outstanding questions remain regarding measurements of the epidemic trajectory and epidemic control strategies given heterogeneous transmission dynamics among the different susceptible species coexisting on the same premises (9, 16).

Here, we developed a multi-host, single-pathogen, multiscale model designed to capture the dynamics of various transmission patterns across different host species to (i) simulate the spread of FMD disease within the Rio Grande do Sul state in Brazil; (ii) describe the geodesic distances from the initial outbreak to secondary cases; and (iii) compare control action strategies, including emergency vaccination, depopulation, various restrictions of between-farm movements, and surveillance activities within control zones, taking into account the initial number of infected farms at the onset of control measures.

## 2 Materials and methods

### 2.1 Data sources

#### 2.1.1 Population data

A comprehensive dataset was compiled from official records of 355,676 farms registered in Rio Grande do Sul, Brazil (19) hosted in the Agricultural Defense System (SDA) (20). The dataset encompassed the number of animals per farm individually for cattle, buffalo, swine, sheep, and goats. Following stringent criteria, 70,853 premises were excluded due to missing geographical coordinates, instances without animal stock, and the absence of incoming and or outgoing movements during the study period spanning from August 24, 2022, to August 24, 2023. Consequently, the final dataset comprised 284,823 farms with accurate and reliable information. To simplify the analysis, population, and movement data from cattle and buffalo farms were merged into a single category denoted as “bovines.” Similarly, sheep and goats were classified as “small ruminants.” The total number of farms by category was as follows: 243,047 bovine farms, 80,664 swine farms, and 41,831 small ruminant farms. Of the total farms, 97,828 raised more than one host species. Supplementary Figure S1 presents farm-level population distribution, and Supplementary Figure S2 presents the geographical farm density distribution.

#### 2.1.2 Birth and death

Producers are required to disclose to SDA their total number of animals born alive and the number of deaths, including those due to natural causes, at least once a year. Here, we collected data on birth and death from SDA, comprising 273,787 individual records associated with births and 268,790 deaths. The daily births and deaths, categorized by species, are depicted in Supplementary Figure S3.

#### 2.1.3 Movement data

From August 24, 2022, to August 24, 2023, 763,448 unique between-farm and from farm-to-slaughterhouse movements were recorded and collected from the SDA centralized traceability database. Upon evaluation of the movement data, 106,481 records (13.9%) were removed due to various reasons: (a) lacking origin or destination identifications; (b) zero animals moved; (c) exact origin and destination premises; and (d) movements from or to premises not registered in the population data or to premises outside the state of Rio Grande do Sul. Ultimately, 413,939 unique between-farm and 243,028 slaughterhouse movements were analyzed. The daily farm-to-farm and farm-to-slaughterhouses movements, categorized by species, are depicted in Supplementary Figure S3.

### 2.2 Outbreak simulation

Rio Grande do Sul has over two hundred thousand farms, of which a sample was drawn and used as initial infected premises. Our sample was multistage and stratified, using the number of farms and species by municipality (21). The sample size was determined considering a prevalence of 50%, with a 95% level of significance and a margin of error of 1.1%, resulting in a total of 10,294 farms (Supplementary Figure S4). Our model simulation was carried out in two steps: First, FMD was seeded randomly via one infected animal into sample farms between August 24, 2022, and August 24, 2023. For farms with multiple species, for instance, farms with bovine, swine, and small ruminants, FMD was seeded into bovine; for farms with swine and small ruminants, FMD was seeded into swine, and for farms with cattle and small ruminants, FMD was seeded via one infected bovine. We assumed that all animals were susceptible to FMD, as the annual vaccination campaign in Rio Grande do Sul had been suspended since May 2021 (22).

### 2.3 Model formulation

**Figure 1:**
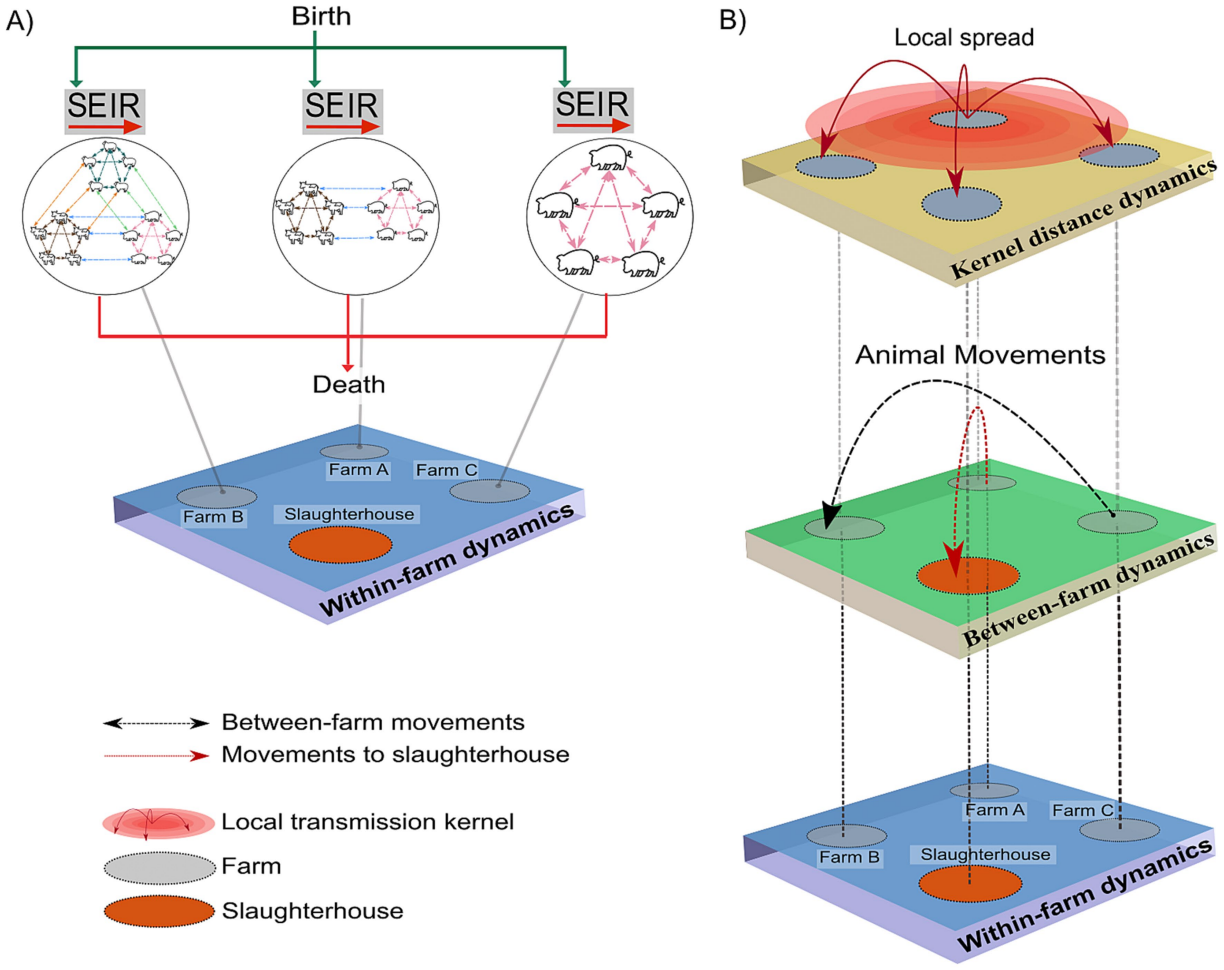
Schematic of state transitions during within-farm and between-farm dynamics. **(A)** Within-farm dynamics: green arrows indicate the introduction of animals (births) into the susceptible (S) compartment at farm *i* at time *t*. Each circle indicates a farm with single or multiple species with specific host-to-host transition parameters (*σ*, *γ*); dashed lines represent interactions within and between host species. The red arrows represent the removal of animals (deaths) regardless of infection status. **(B)** Between-farm dynamics: the layer represents the number of animals moved (batches; *n*) from the farm of origin (*i*) to a destination farm (*j*) at time t (indicated by the black dashed arrows). Animals moved to the slaughterhouse were removed from the simulation regardless of their infection status and are indicated by red dashed arrows. The kernel distance dynamics represent the spatial transmission distances.

We implemented a multi-host, single-pathogen, coupled multiscale model to simulate FMD epidemic trajectories (23) and subsequently applied countermeasures actions. The model led to the development of an R and Python package, entitled “MHASpread: A multi-host animal spread stochastic multilevel model” (version 0.3.0) more details can be consulted in https://github.com/machado-lab/MHASPREAD-model. MHASpread allows the explicit specification of species-specific transmission probabilities and latent and infectious periods of a disease that infects multiple species. The within-farms level includes births and deaths for each species. The between-farm level consists of the entry and exit of animals due to between-farm movements and movements to slaughterhouses (Figure 1).

#### 2.3.1 Within-farm dynamics

For the within-farm dynamics, we assume populations were homogeneously distributed. Species were homogeneously mixed in farms with at least two species, meaning that the probability of contact among species was homogeneous regardless of/when species were segregated by barns (e.g., commercial swine farms are housed in barns with limited changes of direct contact with cattle). The within-farm dynamics consist of mutually exclusive health states (i.e., an individual can only be in one state per discrete time step) for animals of each species (bovines, swine, and small ruminants). These health states (hereafter, “compartments”) include susceptible $\left(S\right)$ , exposed $\left(E\right)$ , infectious, $\left(I\right)\text{,}$ and recovered $\left(R\right)$ , defined as follows:

- Susceptible: animals that are not infected and are susceptible to infection.
- Exposed: animals that have been exposed but are not yet infected.
- Infectious: infected animals that can successfully transmit the infection.
- Recovered: animals that have recovered and are no longer susceptible.

Our model considers birth and death, which is used to update the population of each farm. The total population is calculated as N=S+E+I+R . The number of individuals within each compartment transitions from Sβ→E , 1/→I , 1/γ→R according to the following Equations 1-5:


(1) $$\frac{dS_{i}\left(t\right)}{dt}=u_{i}\left(t\right)-v_{i}\left(t\right)-\frac{\beta S_{i}\left(t\right)I_{i}\left(t\right)}{N_{i}}$$

(2) $$\frac{dE_{i}\left(t\right)}{dt}=\frac{\beta S_{i}\left(t\right)I_{i}\left(t\right)}{N_{i}}-v_{i}\left(t\right)E_{i}\left(t\right)-\frac{1}{\sigma }E_{i}\left(t\right)$$

(3) $$\frac{dI_{i}\left(t\right)}{dt}=\frac{1}{\sigma }E_{i}\left(t\right)-\frac{1}{\gamma }I_{i}\left(t\right)-v_{i}\left(t\right)I_{i}\left(t\right)$$

(4) $$\frac{dR_{i}\left(t\right)}{dt}=\frac{1}{\gamma }I_{i}\left(t\right)-v_{i}\left(t\right)$$

Transmission depends on infected and susceptible host species, as reflected by the species-specific FMD transmission coefficient β (Table 1).

**Table 1:** The distribution of each host-to-host transmission coefficient (*β*) per animal^−1^ day^−1^.

Infected species	Susceptible taxon	Transmission coefficient (β), shape and distribution (minimum, mode, maximum)	Reference
Bovines	Bovines	PERT (0.18, 0.24, 0.56)	Calculated from the 2000–2001 FMD outbreaks in the state of Rio Grande do Sul (22)
Bovines	Swine	PERT (0.18, 0.24, 0.56)	Assumed
Bovines	Small ruminants	PERT (0.18, 0.24, 0.56)	Assumed
Swine	Bovines	PERT (3.7, 6.14, 10.06)	Assumed (60)
Swine	Swine	PERT (3.7, 6.14, 10.06)	(60)
Swine	Small ruminants	PERT (3.7, 6.14, 10.06)	Assumed (60)
Small ruminants	Bovines	PERT (0.044, 0.105, 0.253)	Assumed (61)
Small ruminants	Swine	PERT (0.006, 0.024, 0.09)	(62)
Small ruminants	Small ruminants	PERT (0.044, 0.105, 0.253)	(61)

Births are represented by the number of animals born alive $u_{i}\left(t\right)$ that enter the S compartment on the farm i at the time t according to the day-to-day records; similarly, $v_{i}\left(t\right)$ represent the exit of the animals from any compartment due to death at the time t . The transition from E to I is driven by 1/σ , and the transition from I to R is driven by 1/γ ; these values are drawn from the distribution generated from each specific species according to the literature (Table 2). The dynamics of within-farms are depicted in Figure 1.

**Table 2:** The within-farm distribution of latent and infectious FMD parameters for each species.

FMD parameter	Species	Mean, median (25th, 75th percentile) in days	Reference
Latent period, σ	Bovines	3.6, 3 (2, 5)	(63)
	Swine	3.1, 2 (2, 4)	(63)
	Small ruminants	4.8, 5 (3, 6)	(63)
Infectious period, γ	Bovines	4.4, 4 (3, 6)	(63)
	Swine	5.7, 5 (5, 6)	(63)
	Small ruminants	3.3, 3 (2, 4)	(63)

The time unit is days.

#### 2.3.2 Kernel transmission dynamics

**Figure 2:**
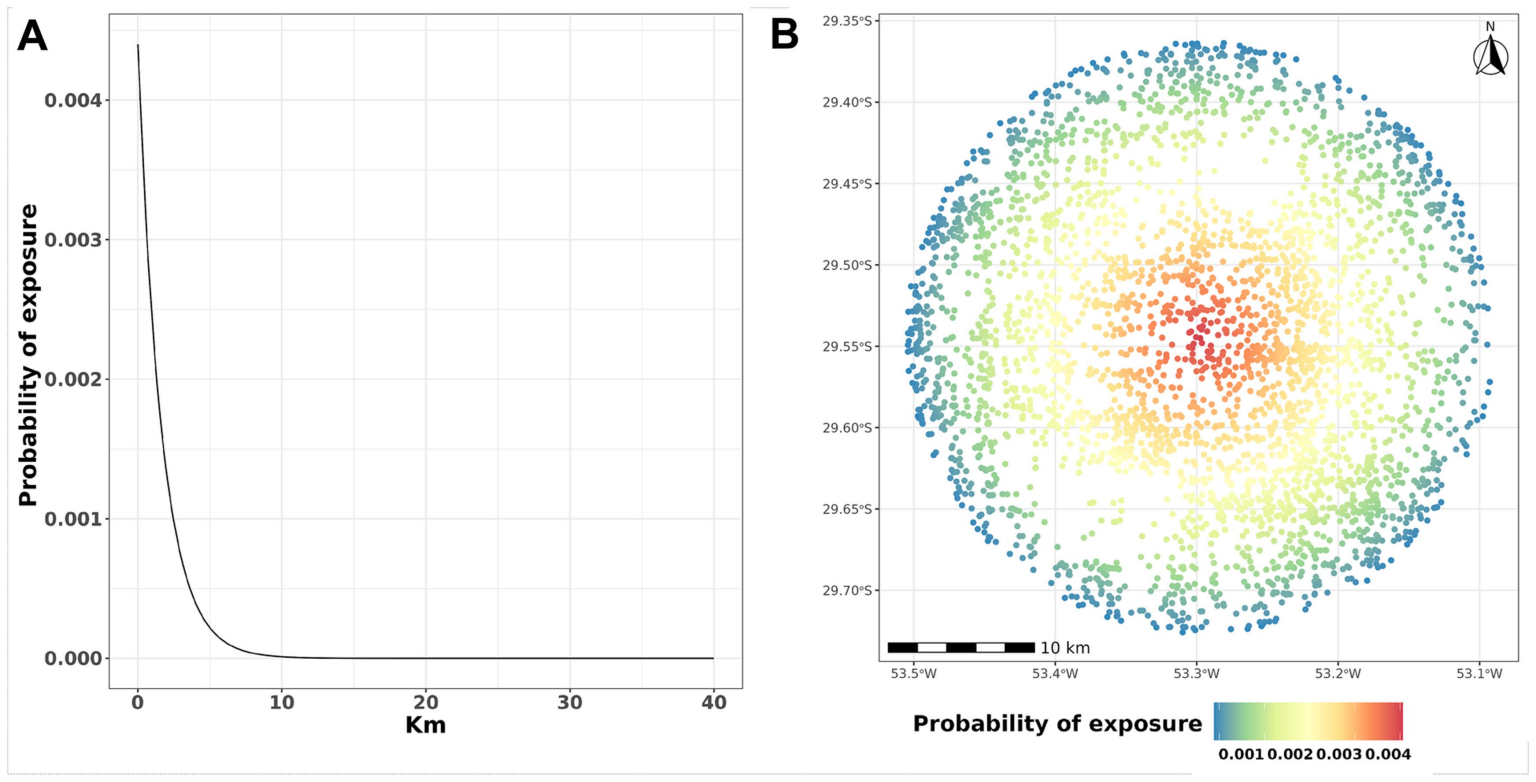
Probability of exposure and distances. **(A)** The y-axis represents the probability of exposure *PE* while the x-axis represents the distance in km. **(B)** Representation of farm locations. The color of the dot represents the probability of *PE* exposure; in this example, the infected farm is located in the center of the radius.

Spatial transmission can occur through various mechanisms, including airborne transmission, contact between animals over fence lines, and the sharing of equipment between farms (24, 25). In this model, we included all these effects by fitting local spread using a spatial transmission kernel. This kernel assumes that the likelihood of transmission decreases as the distance between farms increases, with transmission beyond 40 km not being considered. The probability PE at time t describes the likelihood that a farm becomes exposed and is calculated as follows:


(5) $$PE_{j}\left(t\right)=1-\underset{i}{\prod }\left(1-\frac{I_{i}\left(t\right)}{N_{i}}\varphi e^{-\alpha d_{ij}}\right)$$

where j represents the uninfected population and $ad_{ij}$ represents the distance between farm j and infected farm i , with a maximum of 40 km. Given the extensive literature on distance-based FMD dissemination and a previous comprehensive mathematical simulation study (26), distances above 40 km were not considered. Here, $1-\frac{I_{i}\left(t\right)}{N_{i}}\varphi e^{-\alpha d_{ij}}$ represents the probability of transmission between farms i and j scaled by infection prevalence of farm i , $\frac{I_{i}}{N_{i}}$ , given the distance between the farms in kilometers (Figure 2). The parameters φ and *α* control the shape of the transmission kernel; φ=0.044 which is the probability of transmission when $d_{ij}=0$ , and α=0.6 control the steepness with which the probability declines with distance (24, 25). The exposure probability over distance is depicted in Figure 2.

### 2.4 FMD spread and control actions

We first simulated an initial silent spread over ten days. This procedure yielded a wide range of initial outbreak scenarios, depicted in Figure 2, before implementing control actions. Next, we outline four different control actions scenarios considered outlined by the Brazilian Ministry of Agriculture and Livestock (27).

The baseline control scenario named hereafter as “*base*”: The following measures are considered: (i) depopulation of infected farms, (ii) emergency vaccination of all farms in the infected and buffer zones, (iii) a 30-day animal movement standstill, and (iv) the establishment of three distinct control zones around infected farms, with radii of 3 km (infected zone), 7 km (buffer zone), and 15 km (surveillance zone) (see Supplementary Figure S5), in which control actions vary as described below. These measures aim to prevent the further spread of the disease by enforcing biosecurity protocols and conducting regular inspections of animals and farms within these zones.

Depopulation of infected farms involves the removal of all animals from farms located within the infected zone(s). The daily depopulation capacity was set to four farms for this study, which was aligned with the maximum capacity observed in Rio Grande do Sul (personal communication with Dr. Fernando Groff). Farms with higher animal populations were prioritized for depopulation. Once depopulated, these farms are no longer considered in the simulation. If the daily depopulation capacity was insufficient to cover all identified infected farms within a day, those farms were scheduled for depopulation on the following day or as soon as possible, respecting the maximum capacity constraints.

#### 2.4.1 Emergency vaccination

Bovine farms are vaccinated within infected and buffer zones. The daily maximum capacity was ten farms in the infected zone(s) and ten farms in the buffer zone(s) (personal communication Dr. Fernando Groff.) We simulated the delay in starting vaccination, set to 7 days post-FMD detection. Farms not vaccinated within a day due to the limited vaccination capacity were vaccinated on the subsequent day(s). Here, we do not assume any particular type of vaccine or FMD sorotype. Additionally, vaccine effectiveness was 90% in 15 days (28, 29). For details about the implementation of emergency vaccination, see Supplementary material control actions.

#### 2.4.2 Traceability

We utilized contact tracing to identify farms that had direct contact with infected farms within the past 30 days. These farms underwent surveillance, including clinical examination and detection. Farms testing positive during traceback were categorized as detected infected farms.

#### 2.4.3 Movement standstill

A 30-day restriction on animal movement across all three control zones was implemented, prohibiting any incoming or outgoing movements. The control zones were lifted, and a standstill was maintained until depopulation was complete.

We assumed that 10% of the infected farms were identified when control measures began, specifically after ten days of initial spread from the introduction of the index case. For example, if there were 100 infected farms, only ten would be detected. If the calculated number of detected farms falls below one, we will round up to one detected farm. The detection parameter was set arbitrarily due to the lack of empirical data on the percentage of infected farms at which the surveillance system can reliably detect cases. After the first detection, the detection rate in the subsequent days is influenced by two primary factors: the total number of farms within the control zones and the number of infected farms. Notably, when there are fewer farms under surveillance but a higher number of infected farms, the likelihood of detection increases. Additionally, infected farms located outside the control zones are also included in the pool of farms subject to detection. For more details, refer to Supplementary Figure S6.

#### 2.4.4 Alternative control scenarios

*Base x 2*: In this scenario, the daily number of vaccination increased to 40 farms and the depopulation to eight farms. *Base x 3:* This scenario differed to included 60 farms vaccinated daily and 12 farms depopulated. *Depopulation*. This scenario differed from the baseline control scenario by increasing the depopulation of infected farms to 12, and vaccination was not used.

### 2.5 Model outputs

Our simulations tracked the number of animals in each health compartment and the number of infected farms at each time step. The epidemic trajectories were used to calculate the geodetic distances in km between the seeded index infections and the subsequent infections. In addition, we determined the probability of distance-dependent transmission by calculating the cumulative empirical spatial distribution. We utilized a generalized additive (GAM) model to plot the relationship between the number of infected farms and the epidemic duration across different scenarios, as well as a one-way analysis of variance (ANOVA) with a Tukey post-hoc test to compare scenarios. This enabled us to explore potential nonlinear relationships between the variables, effectively capturing complex patterns that might exist in the data. In addition, a mixed-effects regression model was fitted to describe the relation between days working on control action and the initial number of infected farms controlled by each scenario.

### 2.6 Sensitivity analysis

We used a combination of Latin hypercube sampling (LHS), developed by McKay (30), and the partial rank correlation coefficient (PRCC) technique to perform a local sensitivity analysis. LHS is a stratified Monte Carlo sampling method without replacement that provides unbiased estimates of modeling output measures subject to combinations of varying parameters. The PRCC approach can be used to classify how output measures are influenced by changes in a specific parameter value while linearly accounting for the effects of other parameters (31). As input model parameters, we selected the following categories and interspecies interactions: *β* bovine to bovine, β bovine to swine, β bovine to small ruminants, *σ* bovine, *γ* bovine, β swine to swine, β swine to bovine, β swine to small ruminants, σ swine, γ swine, β small ruminants to small ruminants, β small ruminants to bovine, β small ruminants to swine, σ small ruminants, and γ small ruminants. In total, 15 parameters were used to classify the monotone relation of infection status with our input variables to classify model sensitivity. The inputs include one farm where the initial conditions varied across 10,000 simulations over the LHS space. A positive PRCC indicates a positive relationship with the number of infected animals, whereas a negative PRCC indicates an inverse relationship with the number of infected animals; however, the magnitude of PRCC does not necessarily indicate the importance of a parameter (32).

### 2.7 Software

The language software used to develop the MHASpread model and create graphics, tables, and maps was R v. 4.1.1 (33) and Python v. 3.8.12, R utilizing the following packages: sampler (34), tidyverse (35), sf (36), brazilmaps (37), doParallel (38), lubridate (39) and Python v. 3.8.12 with the following packages: Numpy (40), Pandas (41), and SciPy (42). This model is available in both R and Python versions.

## 3 Results

### 3.1 Initial spread and detection

**Figure 3:**
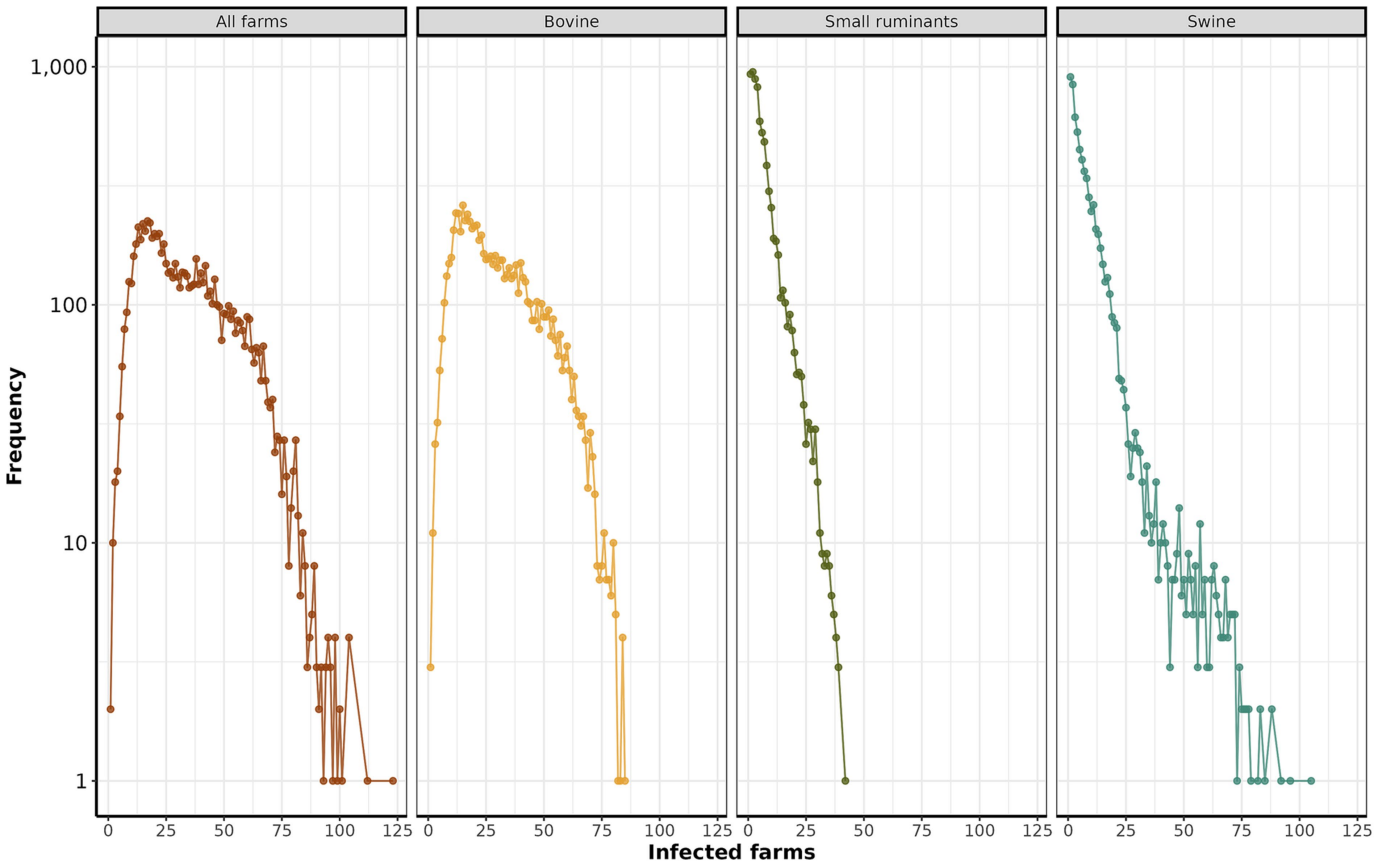
Distribution of the initial infected farms. The y-axis depicts the logarithm (base 10) of the frequency of infected farms across all simulations. The x-axis represents the number of infected farms.

Initially, we explore the variation in initial infection trends within the first ten days (Figure 3). The median number of infected farms was 52.5 (IQR: 26.75 to 78.25, maximum 123), of which the majority were swine farms with a median of 43.5 (IQR: 22.25 to 64.75, maximum 105), compared to bovine 43 (IQR: 22 to 64, maximum 85) and small ruminants with 20.5 (IQR: 10.75 to 30.25, maximum 42).

### 3.2 Distances from the initial outbreak

**Figure 4:**
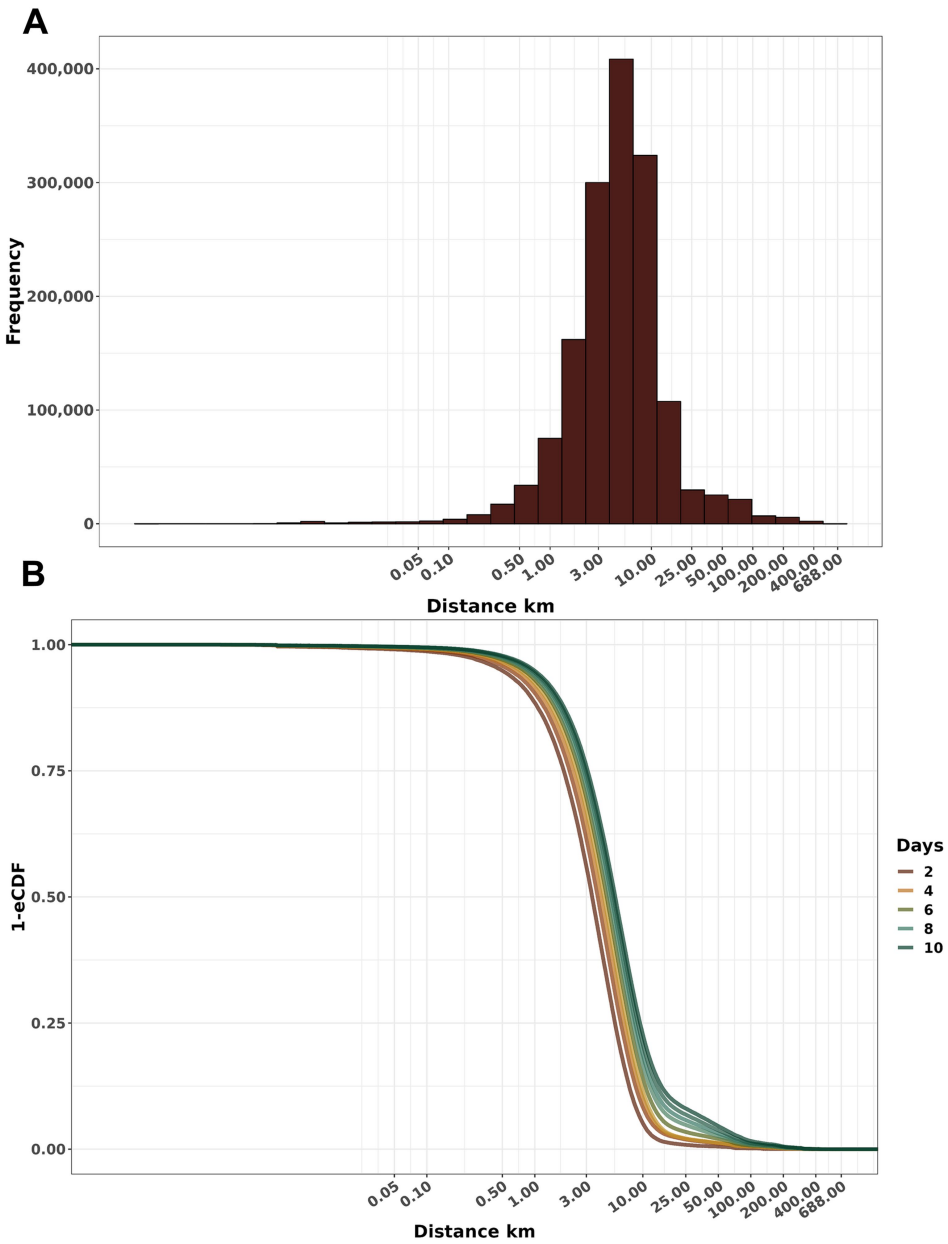
Distribution of the cumulative distance of dissemination. **(A)** Frequency histogram of secondary infections at varying distances from the seeded infection. **(B)** Empirical cumulative distribution function (1-ECDF) probability of infection according to distances from the initial outbreak within the days after the disease introduction. Both x-axes are a log10 scale.

Of the 284,396 unique simulated FMD events, the distance from seeded infection to the secondarily infected farm within the first ten days exhibited a median of 4.78 km (IQR: 2.64 Km to 7.98 Km, maximum 6.88 Km) (Figure 4A). Furthermore, we observed a linear increase in the distance to which FMD disseminated (Figure 4B).

### 3.3 Effectiveness of control measures

**Figure 5:**
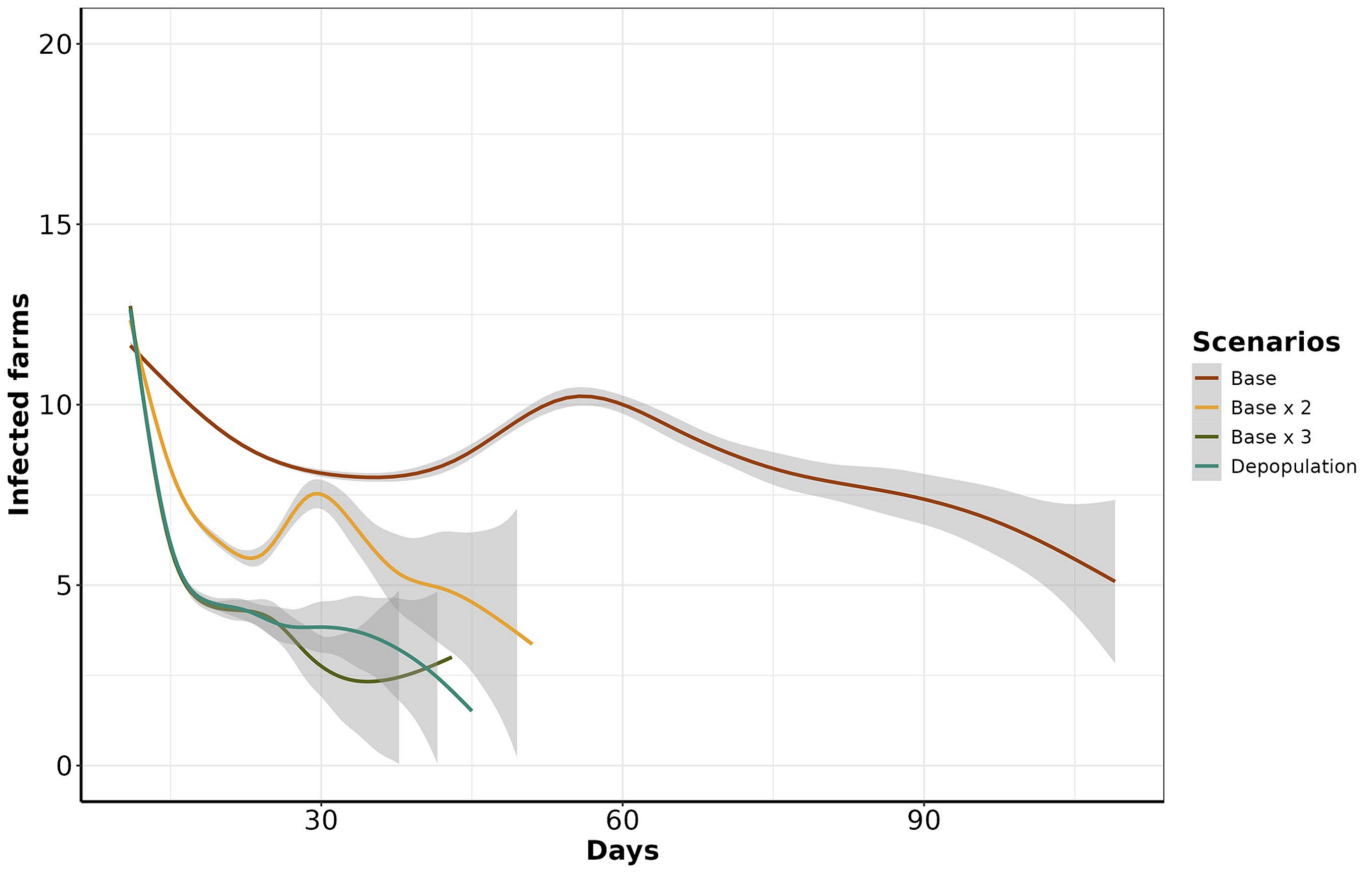
Estimated number of infected farms from day 11 to 120. The y-axis represents the number of infected farms, while the x-axis represents the day of simulation of control actions. The color line corresponds to each scenario.

All control scenarios were effective in eliminating all outbreaks within 120 days of the start of control measures. However, effectiveness was significantly different (ANOVA, *p*-value <0.05), except when we compared *depopulation* with the *base x 3* scenario. In general, the most effective alternative scenarios were *base x 3* and *depopulation*. The most notorious differences in means of infected farms by scenario were between the *base* and *depopulation* scenarios with mean differences of 2.36 (95% CI: 2.22 to 2.49), followed by *base x 3* and *base* with 2.26 (95% CI: 2.13 to 2.39) and *base x 2* and *base* with 1.35 (95% CI: 1.22 to 1.49) (Supplementary Figure S8). In addition, we used a generalized additive model (GAM) to visualize the course of simulated epidemics over time. Notably, scenario *base x 3* consistently exhibited lower prevalence over time when benchmarked with *depopulation*, *base x 2*, and *base* scenarios (Figure 5).

#### 3.3.1 Control actions duration

**Figure 6:**
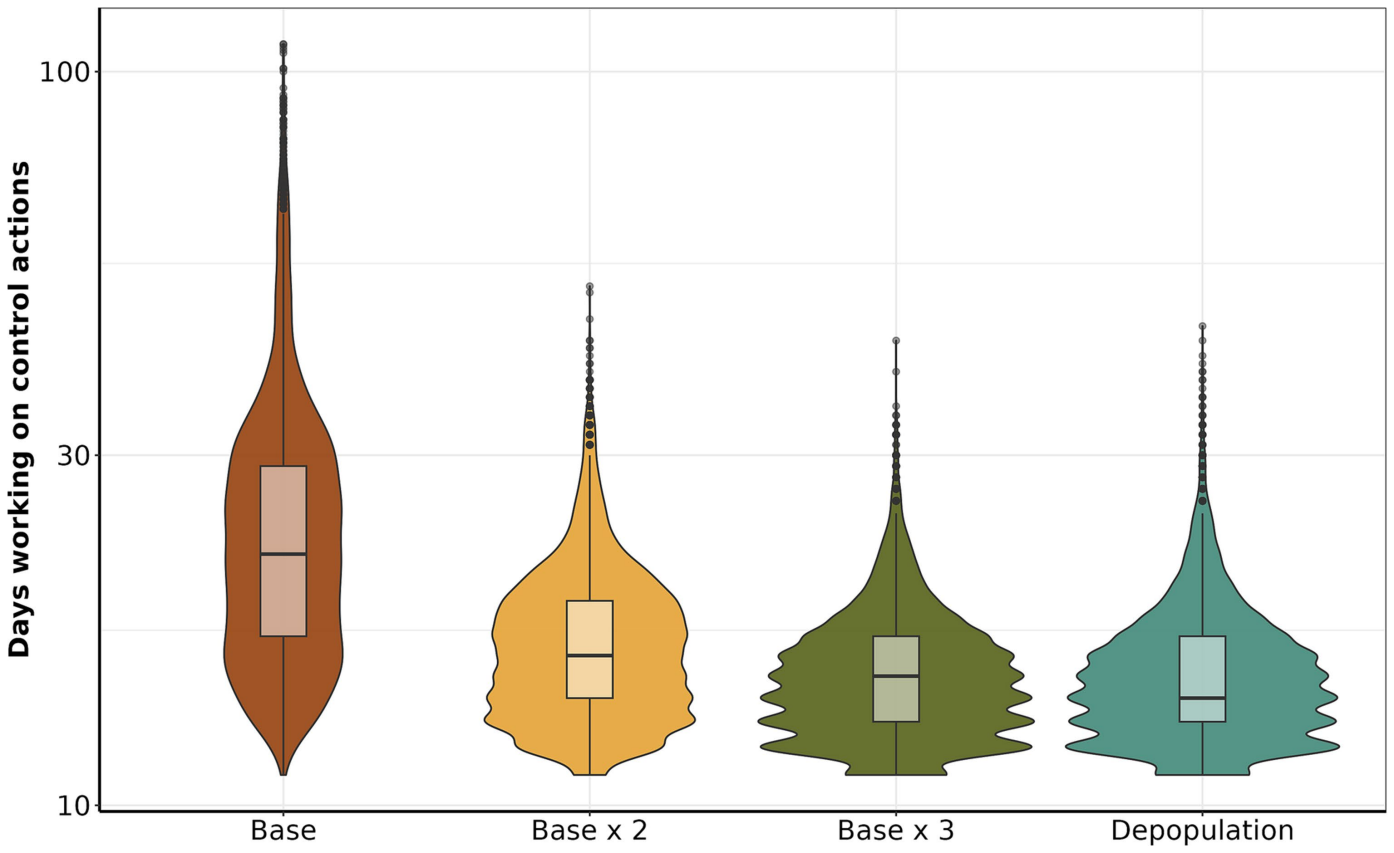
Comparison of the number of days working on control actions. The y-axis shows the total number of days dedicated to each control action, the x-axis presents the scenarios.

The median number of days of control actions implemented for the *base* scenario was 22 (IQR: 17 to 29, maximum 109). While *base x 2* had a median of 16 (IQR: 14 to 19, maximum 51), *base x 3* with a median of 15 (IQR: 13 to 17, maximum 43) *depopulation* had 14 days (IQR: 13 to 17, maximum 45) (Figure 6). In addition, we describe the similarities and disparities between the mean number of days control actions were active, meaning at least one outbreak response action was still ongoing. The comparison between *depopulation* and *base*, *base x 3* and *base*, and *base x 2* and *base* revealed substantial disparities in the group means: -9.77 (95% CI: to 10.04 to −9.49), −9.83 (95% CI: −10.10 to −9.56), and − 8.09 (95% CI: −8.36 to −7.81), respectively. The complete statistics are depicted in Supplementary Figure S9. Our finding indicates a positive relationship between time working in control action and the number of initially infected farms. We found a linear relationship in which, on average, for each additional infected farm at the beginning of the control actions, the number of days working on control actions is expected to increase by approximately 1.59 days (GLM, *p*-value <0.05).

#### 3.3.2 Vaccination

**Figure 7:**
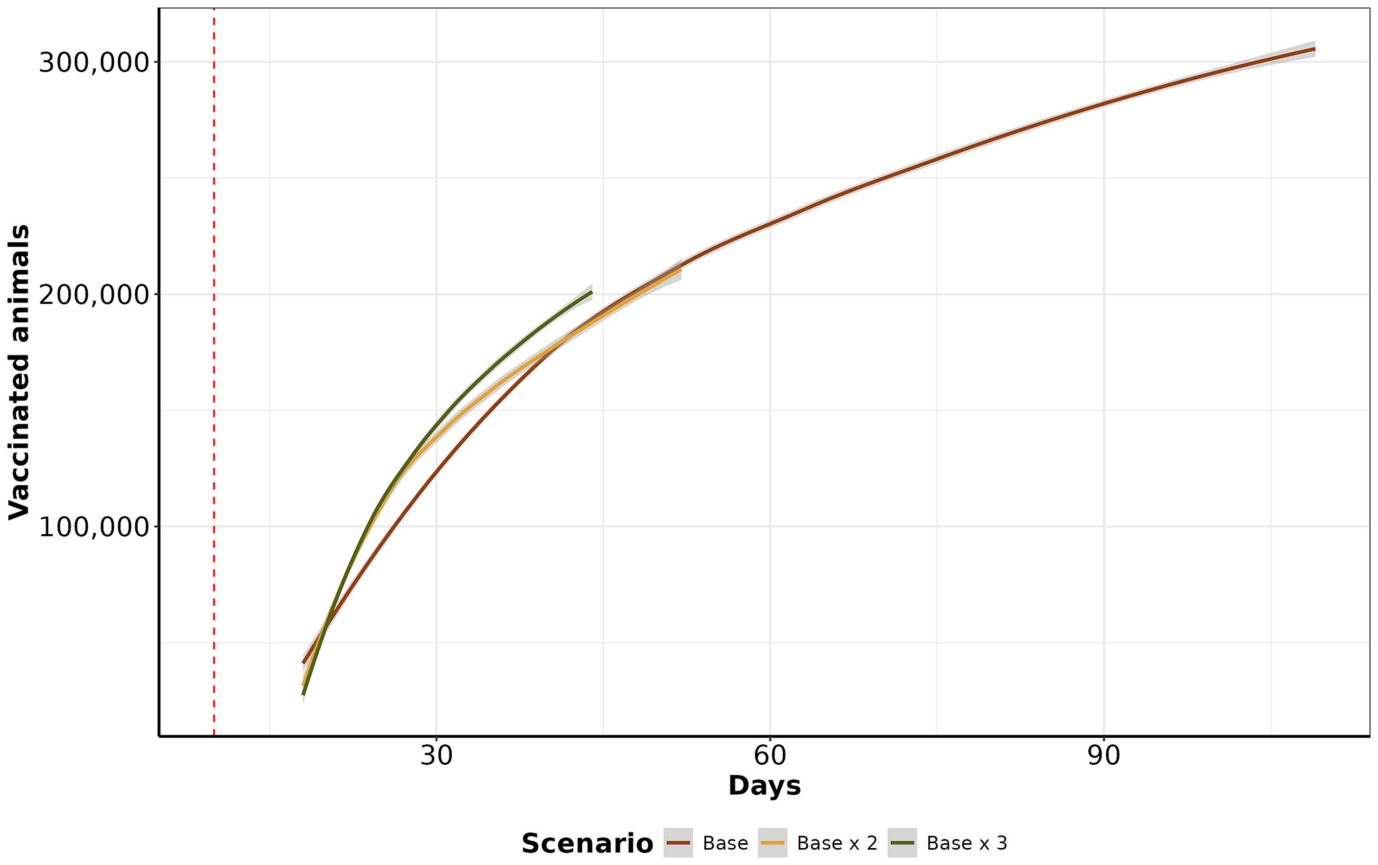
Vaccination curve by scenario. The y-axis represents the cumulative average of vaccinated animals per day, in the log10 scale. The x-axis shows the day of vaccination. The red dashed line represents when control actions were initiated after 15 days of initial the other control actions.

In the *base* scenario, the daily median of vaccinated animals was 1,928 (IQR: 1,562 to 3,567, maximum: 20,740). In the *base x 2* scenario, the median increases to 3,959.32 (IQR: 3,067.75 to 5,865.56, maximum: 25,877). Similarly, in the *base x 3* scenario, the median increases to 5,947 (IQR: 4,157 to 8,384, maximum: 25,006). In the initial 30 days, there was a significant increase in the number of vaccinated animals, and after that, the amount of vaccine continued to increase on a reduced step demand (Figure 7).

#### 3.3.3 Depopulation

**Figure 8:**
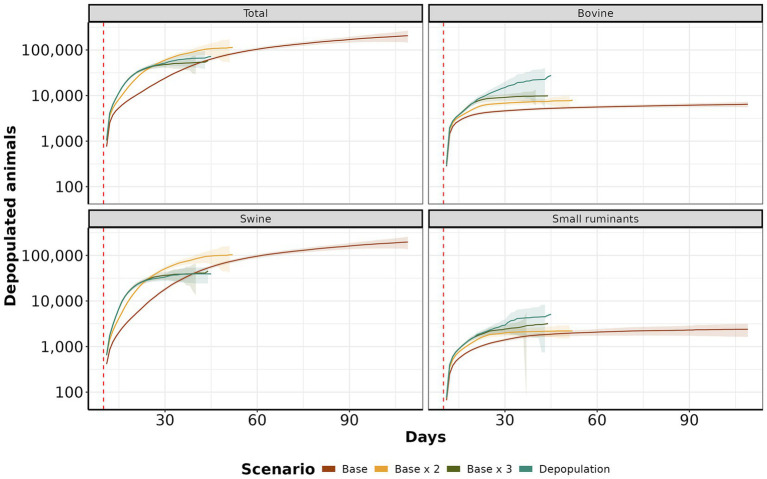
Depopulation curves by scenario. The y-axis shows the daily cumulative average of depopulated animals. The x-axis represents the day. The red dashed line color represents each control scenario.

We analyzed the daily average number of depopulated animals over time. Scenario *base x 2* and *depopulation* showed the highest cumulative mean with 3,071 (IQR: 1,767 to 3,768, maximum: 4,120) and 2,159.09 (IQR: 1,314 to 3,000, maximum: 3,830), respectively. Following closely were the *base* and *base x 3* scenarios, with means of 2,139 (IQR: 1,798 to 2,541, maximum: 3,039) and 1,151.93 (IQR: 500 to 2,799, maximum: 4,398), respectively. The *depopulation* scenario consistently showed the highest count of affected animals, followed by *base x 3*, *base x 2*, and *base*, particularly for bovine and small ruminants (Figure 8). However, in the case of swine, scenarios *base* and *base x 2* exhibited a higher incidence than other species.

### 3.4 Sensitivity analysis

We evaluated the sensitivity of 15 model parameters with weights ranging from −0.86 to 0.72; this sensitivity indicated a limited influence of model parameters on the number of simulated secondary infections. Specifically, the latent period σ had a negative impact on the number of secondary infections, and the Infectious period γ had a positive influence on the number of secondary infections; both σ and γ have significant results (*p*-value <0.05) overall simulated species. The complete sensitivity analysis results are depicted in Supplementary Figure S10.

## 4 Discussion

This study aimed to develop an FMD multiscale, multispecies stochastic model that explicitly incorporates species-specific transmission interaction. Our model was used to simulate the spread of FMD among cattle, buffalo, swine, and small ruminants of Rio Grande do Sul, Brazil, and to examine the effectiveness of countermeasure scenarios. Bovine farms were the most infected species, followed by swine and small ruminants, mostly because of the higher number of cattle farms, and the connectivity of the swine contact network. Most secondary infections spread within 25 km, showing that disease transmission by proximity plays an important role in the spread dynamics. Our simulations demonstrated that tripling the number of daily depopulated and the vaccination eliminated epidemic trajectories within 15 days, which required 5,947 animals to be vaccinated and the depopulation of 2,139 animals.

Within ten days of introduction, the range of infected farms varied from 1 to 123, with the majority of simulations (90.12%) resulting in fewer than 50 infected farms. Our study also revealed that when FMD infected swine, the epidemic sizes were significant (Figure 3). This risk is of particular relevance to areas of dense swine populated with commercial swine production, which are typically vertically integrated, which means such farms move a significant number of swine facilitation long-distance spread (43–45). Our study demonstrated that the number of farms initiating control actions has a linear impact on the duration of these actions, regardless of the implemented scenario. Specifically, each additional infected farm extended control actions by an average of 1.6 days. Consequently, enhancing the sensitivity of foreign animal disease detection is crucial for optimizing the effectiveness of control strategies (46). Therefore, we argue that improving the timing of detections and optimizing the response and management of outbreaks are pivotal to ensuring effective control. The scenario *base x 3* demonstrated the best performance compared to the other proposed scenarios, requiring a median of 15 days to eliminate the outbreak (Figure 4). For comparison, the *base* scenario had a median duration of 22 days, and the *base x 2* scenario had a median duration of 16 days. Due to the large number of vaccines administered in the *base x 3* scenario, averaging 5,947 per day, compared to 1,928 per day in the *base* scenario and 3,959 per day in the *base x 2* scenario. Finally, the depopulation of 12 farms daily was successful in mitigating outbreaks; however, this scenario poses a significant challenge for official services.

Emergency vaccination presents an alternative to preemptive culling policies but may also limit the accuracy of surveillance systems in detecting infected farms, as it may mask clinical signs (47, 48). When examining the median number of vaccinated animals at the end of the control action scenarios, the *base* scenario had the highest median of vaccinated animals at 305,105, followed by *base x 2* with 210,786 and *base x 3* with 202,471 vaccinated animals. Interestingly, despite the higher vaccination rates, the final average number of vaccinated animals was lower in scenarios with increased vaccination rates. This occurs because increasing vaccinations reduces the duration of outbreaks, ultimately resulting in fewer doses at the end of control actions. Our findings align with studies from Australia, Canada, New Zealand, and the United States, where emergency vaccination was correlated with a reduction in the number of infected farms and a decrease in outbreak duration (49–53). Examining the cumulative number of depopulated animals, we demonstrated that the *depopulation* scenario was more effective in controlling epidemics than the *base* and *base x 2* scenarios. The primary reason for this effectiveness was the high intensity of farm depopulation per day. Our analysis of the daily average number of depopulated animals over time reveals that the *base x 2* scenario had the highest mean cumulative count, with 3,071 animals culled, followed closely by the *depopulation* scenario, with 2,159 animals. The *base* scenario had a mean of 2,139 animals, while the *base x 3* scenario had 1,151 animals. When examining depopulation by species, the *depopulation* scenario consistently recorded the highest number of culled animals, particularly for bovine and small ruminants. In contrast, the *base* and *base x 2* scenarios revealed a higher prevalence of culled animals in swine than other species. The main reason for this is that prolonged outbreaks tend to affect more pig farms. Despite fewer pig farms than cattle farms, the number of animals per pig farm is significantly higher (Supplementary Figure S1). While depopulating was an effective countermeasure to contain highly contagious diseases like FMD (51, 54, 55), culling healthy animals raises ethical concerns. It also increases economic losses due to reduced production and farmer compensations (56). Conversely, other studies have proposed alternatives to ring depopulation, for instance, Seibel et al. (16) simulated target density strategy and showed its advantages in combating FMD since the number of healthy animals depopulated was lower than traditional total ring depopulation while the time to eliminate the outbreaks were similar. Besides, we emphasize the significance of timing when initiating depopulation for FMD control to prevent a disease outbreak across all farms in the area and potential outward spread (57). Moreover, prolonged delays in culling can lead to recurrent outbreaks in previously controlled areas, and under specific atmospheric conditions, there exists a risk of long-distance airborne spread (55).

### 4.1 Limitations and further remarks

Since there are no recent FMD outbreaks in the study region, we used data from the most recent FMD outbreak in Rio Grande do Sul (2000 and 2001) to extract parameters while utilizing the literature for the remaining parameters. Our sensitivity analysis did not identify any number of infected animals. Thus, our model has an acceptable level of robustness. FMD virulence, infectivity, and transmission can vary among strains (1, 11). Even though the most recent outbreaks in the State of Rio Grande do Sul were serotypes O and A (22), we cannot rule out the possibility that other strains were introduced and exhibited different dissemination patterns. Future work could include transmission scenarios with strains circulating in neighboring countries.

Additionally, other important between-farm transmission routes, such as vehicles and farm-staff movements, which have been previously associated with FMD dissemination (58, 59), were not included in our model. If such indirect contact networks are considered, the results would likely change, and model realism would be improved (51). We assumed 100% compliance with the restriction of between-farm movement from infected farms and farms directly linked to infected farms and the restriction of movement into and from control zones; we also assumed that the disposal of depopulated animals eliminated any possibility of further dissemination. Nevertheless, real-world compliance with the control actions was not examined or considered. Our model can also provide a distribution of expected FMD epidemics for any current or future control actions listed in the Brazilian control and elimination plan (27). Nevertheless, because our results are based on population data and between-farm movement data from Rio Grande do Sul, the interpretation of our findings should not be extrapolated to other regions. However, since the MHASpread model, infrastructure is highly flexible and can be easily extended to other Brazilian states and other countries.

## 5 Conclusion

In summary, we have shown the importance of including species-specific propagation dynamics in FMD transmission models designed to assist decision-makers in planning control and mitigation strategies for FMD. We have shown that a quick response in initiating control actions on a lower number of infected farms is crucial to reduce the necessary duration of control actions. We found that increasing depopulation capacity was sufficient to eliminate outbreaks without vaccination. Eliminating infected or likely-infected animals is an optimal strategy for preventing further epidemics, but culling large numbers of healthy animals raises welfare concerns. Regardless of which species in which FMD was introduced, the median distance over which the disease spread was within 25 km, a finding that could explain the effectiveness of the simulated countermeasures within the control areas used for FMD response. Our model projections, along with the necessary software, are available to local animal health officials. Thus, our model can be used as a policy tool for future responses to FMD epidemics through computer-based preparedness drills and capacity building and during emergency responses to FMD epidemics by providing rules of thumb generated from simulated control scenarios.

## Data Availability

The raw data supporting the conclusions of this article will be made available by the authors, without undue reservation.
